# Ideal sphere-forming culture conditions to maintain pluripotency in a hepatocellular carcinoma cell lines

**DOI:** 10.1186/s12935-015-0240-y

**Published:** 2015-10-09

**Authors:** Seon Ok Min, Sang Woo Lee, Seon Young Bak, Kyung Sik Kim

**Affiliations:** Department of Surgery, Yonsei University College of Medicine, 50-1 Yonsei-ro, Seodaemun-gu, Seoul, 120-752 Korea; Graduate Program of Nano Science and Technology, Graduate School of Yonsei University, Seoul, Korea; Cell Therapy Center, Severance Hospital, Yonsei University College of Medicine, Seoul, Korea

**Keywords:** Neoplastic stem cells, Hep G2 cells, Pluripotent stem cells, Bovine serum albumin

## Abstract

**Background:**

Cancer stem cells (CSCs) constitute 1–2 % of cancer tissue and are a major cause of tumor metastasis and recurrence. The culture environment is important in maintaining CSCs in vitro. Sphere formation is one method of culturing CSCs. In this study, we identified and validated optimal culture conditions for sphere formation in hepatocellular carcinoma cells.

**Methods:**

Huh7 and HepG2 cells were plated in three different media types and were allowed to form spheres. To confirm the pluripotency of sphere cells, the expression of stem cell markers was evaluated. EpCAM, Connexin 32, and Connexin 43 expression was evaluated using reverse transcription-polymerase chain reaction (RT-PCR). The expression of E-cadherin, β-catenin, CD90, and CD133 was evaluated using immunocytochemistry. Flow cytometry was performed to confirm the presence of stem cell markers CD133 and Connexin 32.

**Results:**

Cells maintained in medium containing growth factors ((DMEM(+))GF) showed greater cell proliferation than cells in media with fetal bovine serum (FBS) (DMEM(+)FBS) or without FBS (DMEM(−)FBS). Cells cultured in DMEM(+)FBS medium exhibited greater proliferation after 20 days in culture than cells cultured under the other two conditions. Cells cultured in DMEM(−)FBS medium did not proliferate; therefore, this condition was removed from further analysis. RT-PCR and flow cytometry showed that sphere-forming cells cultured in DMEM(+)GF and DMEM(+)FBS media had similar expression of stem cell markers.

**Conclusion:**

Therefore, growth factor-free medium is an adaptable, efficient, and cost-effective tool for in vitro cultivation of CSCs.

## Background

Cancer stem cells (CSCs) play an important role in the formation, progression, and recurrence of malignant tumors [1]. CSCs comprise 1–2 % of cancer tissue and are particularly resistant to anticancer agents; instead of dying, CSCs become latent and then differentiate into cancer cells again, contributing to metastasis and uncontrolled growth.

The possibility that cancer may originate from stem cells was suggested by Cohnheim [2], and the theory has gained momentum with systematic study [3]. Hepatocellular carcinoma (HCC), the fifth most common cancer in the world, is a malignant tumor that is the third most common cause of cancer-related death [4]. Although there are various treatment methods for HCC, including surgical resection, liver transplant, chemotherapy, and radiation therapy, the cure rate is low, and the recurrence rate is high. CSCs are considered as the factors responsible for the cases of tumor relapse. Liver CSCs have been reported in multiple subtypes of HCC and are considered as the master regulators of HCC initiation, tumor metastasis, and progression [5]. Drug therapies specifically targeting CSCs may be a more efficient cancer therapy for these patients. Therefore, researchers are attempting to identify the characteristics of CSCs using cancer tissues and Huh7 cell lines from patients [6]. Studies have been conducted to elucidate the role of CSCs in liver cancer to reduce the high recurrence rate, but the findings are controversial regarding accurate stem cell markers and cell separation methods. These studies show that the methods to efficiently culture CSCs from cancer cells are lacking, and more effective culture conditions are required.

Sphere-forming culture was first used by Reynold and Weiss to isolate stem cells and has been widely used since [7]. In this technique, cells are attached to an extracellular matrix (ECM) that limits the growth, proliferation, and differentiation of the cells. If cells cannot attach to the ECM and grow in suspension, they die through anoikis [3, 8]. However, cells with properties similar to stem cells are not affected by the ECM and can proliferate only through cell–cell interaction. Using these properties, Reynold and Weiss achieved a free-floating sphere culture, called a neurosphere culture, using stem cells obtained from the human brain [7]. CSCs are similar to stem cells in that the former can differentiate into cancer cells. Cell-surface markers expressed in stem cells such as CD133, CD44, and CD90 are also expressed in liver CSCs [9–11]. Cells growing under sphere-forming conditions also have increased viability. The sphere-forming culture method has been used to proliferate stem cells in solid cancers such as those of the breast and ovary [12]. Under the ECM-attached condition, the survival rate of cancer cells in a sphere-forming culture was higher than that in a monolayer culture. In addition, several stem cell markers such as oct4, ov6, and CD133 were more highly expressed. When cells obtained from sphere-forming culture methods were transplanted into mice, cancer formed more readily than when using an equal number of cells grown in a monolayer [13].

One of the most important components of sphere-forming culture is the optimal culture medium. The culture medium used in monolayer culture is generally different from that used in sphere-forming culture. Sphere-forming culture medium is serum-free and supplemented with several growth factors, including epidermal growth factor (EGF), basic fibroblast growth factor (bFGF), b27 supplement, and others. The concentration of growth factors added is different for different types of cells, and the effect of each growth factor is also distinct. For example, EGF signaling is activated by EGF receptors and is known to play an important role in maintaining pluripotency in neuroglioma stem cells [14]. FGF is known to play an important role in creating tumor spheres and increasing side population which is sub-population of cells that is distinct from the main population on the basis of the markers employed [15]. b27 plays a role in forming tumor spheres and in maintaining the characteristics of sphere-forming cells [16].

There are cancer cells in which the sphere-forming growth of CSCs is possible without the addition of growth factors. Glioblastoma stem cells, for example, are known to proliferate with only autocrine factors even in the absence of external growth factors [17].

In the current study, sphere formation of Huh7 and HepG2 cells was evaluated in culture media with and without growth factors. Culture conditions potentiating optimal sphere formation, while also maintaining the stem cell characteristics of CSCs, were established.

## Results

### Sphere formation in Huh7 and HepG2 cells

**Fig. 1:**
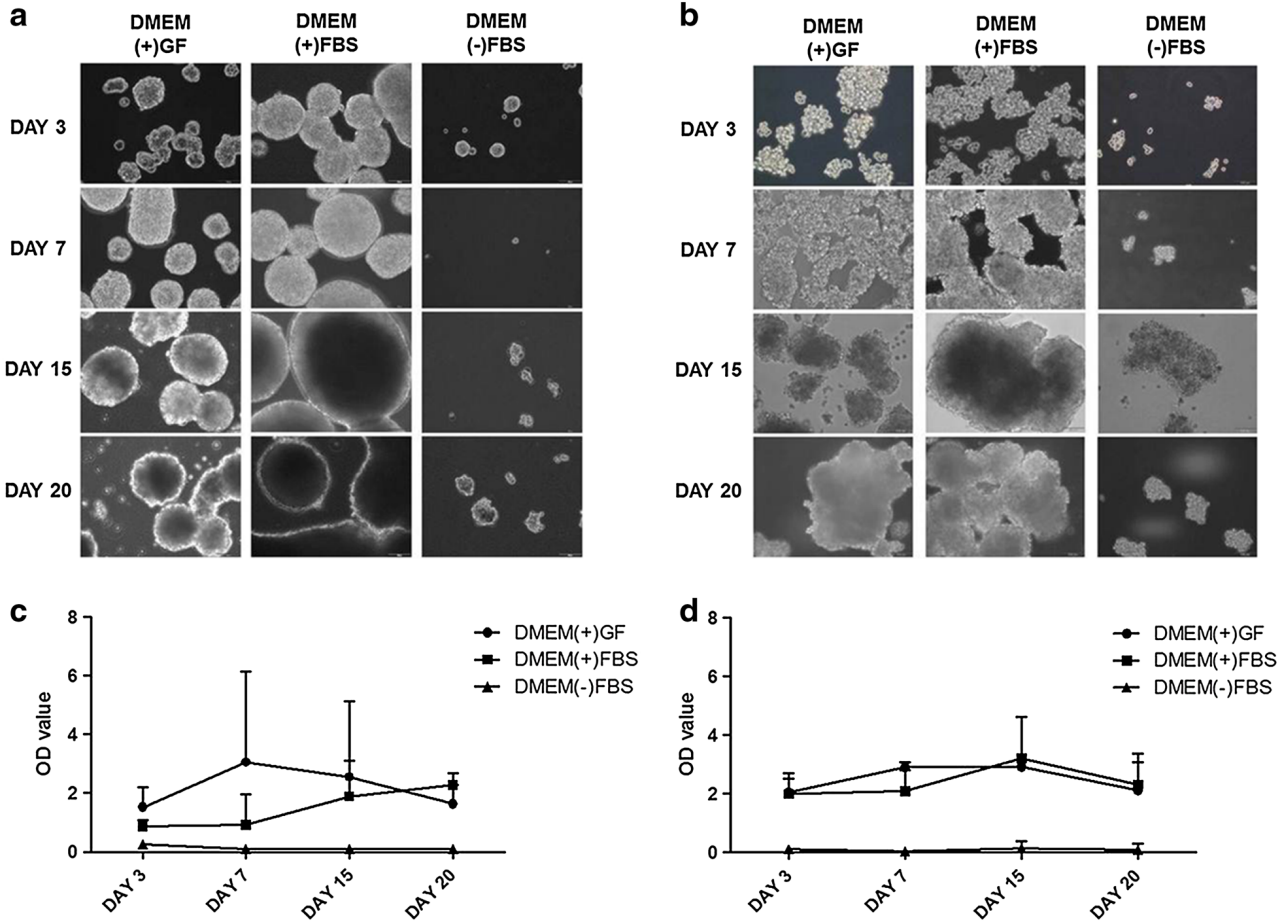
Optical microscopy and proliferation analysis of Huh7 spheres in DMEM(+)GF, DMEM(+)FBS and DMEM(−)FBS medium on days 3, 7, 15, and 20. Hepatocellular carcinoma cell lines, including **a** Huh7 and **b** HepG2, could form the spheres in DMEM(+)GF and DMEM(+)FBS medium. *Scale bar* 100 µm, *scale bar* 100 µm. Huh7 and HepG2 cells were cultured in three media types (DMEM(+)GF, DMEM(+)FBS and DMEM(−)FBS media) and were observed over time. Cells were not adhered to culture plates but grew in suspension and formed spheres. Cells in DMEM(+)GF and DMEM(+)FBS media formed spheres that were similar in size, which increased after day 15. Cells in the DMEM(−)FBS medium died and were excluded from further analyses. The proliferation of sphere-forming cells in DMEM(+)GF, DMEM(+)FBS and DMEM(−)FBS was compared over time. Cells were analyzed on days 3, 7, 15, and 20 using a CCK-8 kit. **c** The Huh7 sphere-forming cells showed a cell proliferation. **d** The HepG2 sphere-forming cells showed a cell proliferation. After 3 days in culture, the proliferation of sphere-forming cells in DMEM(+)GF medium was increased. After 7 days in culture, the proliferation of DMEM(+)GF and DMEM(+)FBS media cultured cells became similar. **b** The HepG2 sphere-forming cells showed a cell proliferation. Cells in DMEM(+)FBS media were increased for 15 days and in DMEM(+)GF media decreased from 7 days

Sphere formation was evaluated in the three types of media, DMEM(+)GF, DMEM(+)FBS and DMEM(−)FBS. Sphere formation began in DMEM(+)GF and DMEM(+)FBS cultured cells on day 5 (data not shown). Cells cultured in DMEM(−)FBS, which lacked FBS and growth factors, did not form spheres and died in culture. The morphology of DMEM(+)GF and DMEM(+)FBS cultured spheres were similar to each other. The sphere-forming cells started to attach to each other on day 7 under both conditions, respectively. The size of DMEM(+)GF and DMEM(+)FBS cultured cells was not increased, but their form was maintained HepG2 sphere-forming cells were similar with Huh7 cells (Fig. 1a, b).

### Analysis of the self-renewal of Huh7 and HepG2 sphere-forming cells

The cell proliferation rates of the DMEM(+)GF and DMEM(+)FBS groups were not significantly different after 3 day, but the proliferation of sphere-forming cells in DMEM(+)GF was significantly increased after 7 days. Increased proliferation rates of sphere-forming cells in DMEM(+)FBS was not observed until day 7 and could be observed through day 15.

After 2 weeks in culture, the proliferation rate of the cells in DMEM(+)GF decreased, but proliferation of the cells in DMEM(+)FBS increased again by day 20. Although in the DMEM(+)GF, the number of HepG2 sphere-forming cells increased until day 7, number of HepG2 sphere-forming cells in DMEM(+)FBS did not increase until day 7 and increased between days 7 and 15. After day 15, the number of HepGe2 sphere-forming cells decreased in both culture conditions. Sphere formation did not occur in the cells in DMEM(−)FBS, and the cells did not proliferate (Fig. 1c, d).

### Expression of cancer stem cell markers in Huh7 sphere-forming cells

**Fig. 2:**
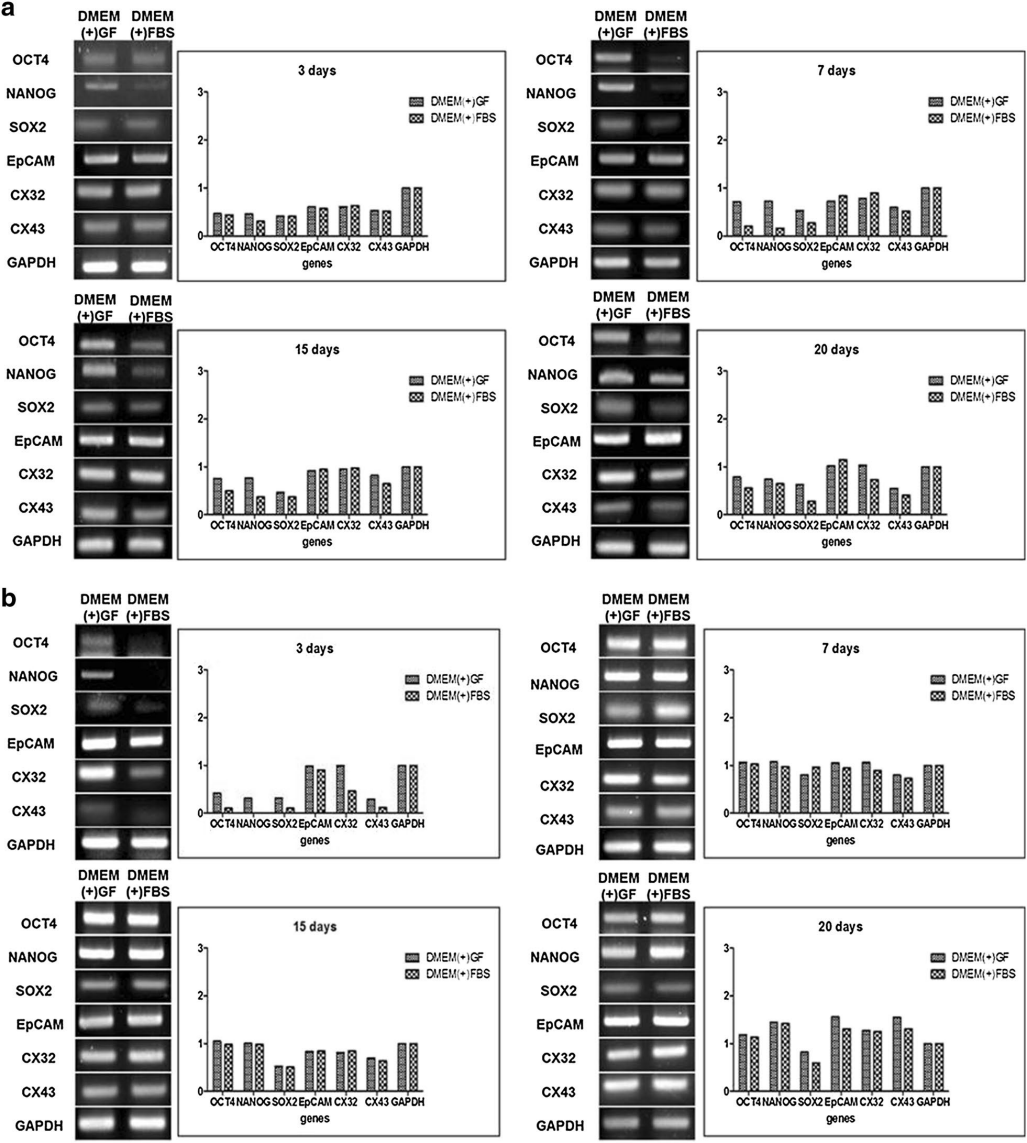
Expression of CSC markers in Huh7 and HepG2 spheres. **a** Huh7 and **b** HepG2 sphere-forming cells. Expression of gene levels were similar between the two groups. The expression of the housekeeping gene GAPDH was used as a loading control. *Cx32* Connexin 32, *Cx43* Connexin 43

Expression of pluripotency and CSC marker genes were measured using RT-PCR. Initially, the expression of pluripotency genes in Huh7 and HepG2 cells were higher in DMEM(+)GF; however, as the culture period progressed, expression of pluripotency genes in DMEM(+)GF and DMEM(+)FBS-cultured cells became similar. The expression of epithelial cell adhesion molecule (EpCAM), a gene expressed in CSCs, was similar between the two groups. The expression levels of Connexin 32 and 43, which are involved in the proliferation and growth of hepatoma cells, were also not significantly different (Fig. 2).

### Expression of CSC surface proteins in Huh7 sphere-forming cells

**Fig. 3:**
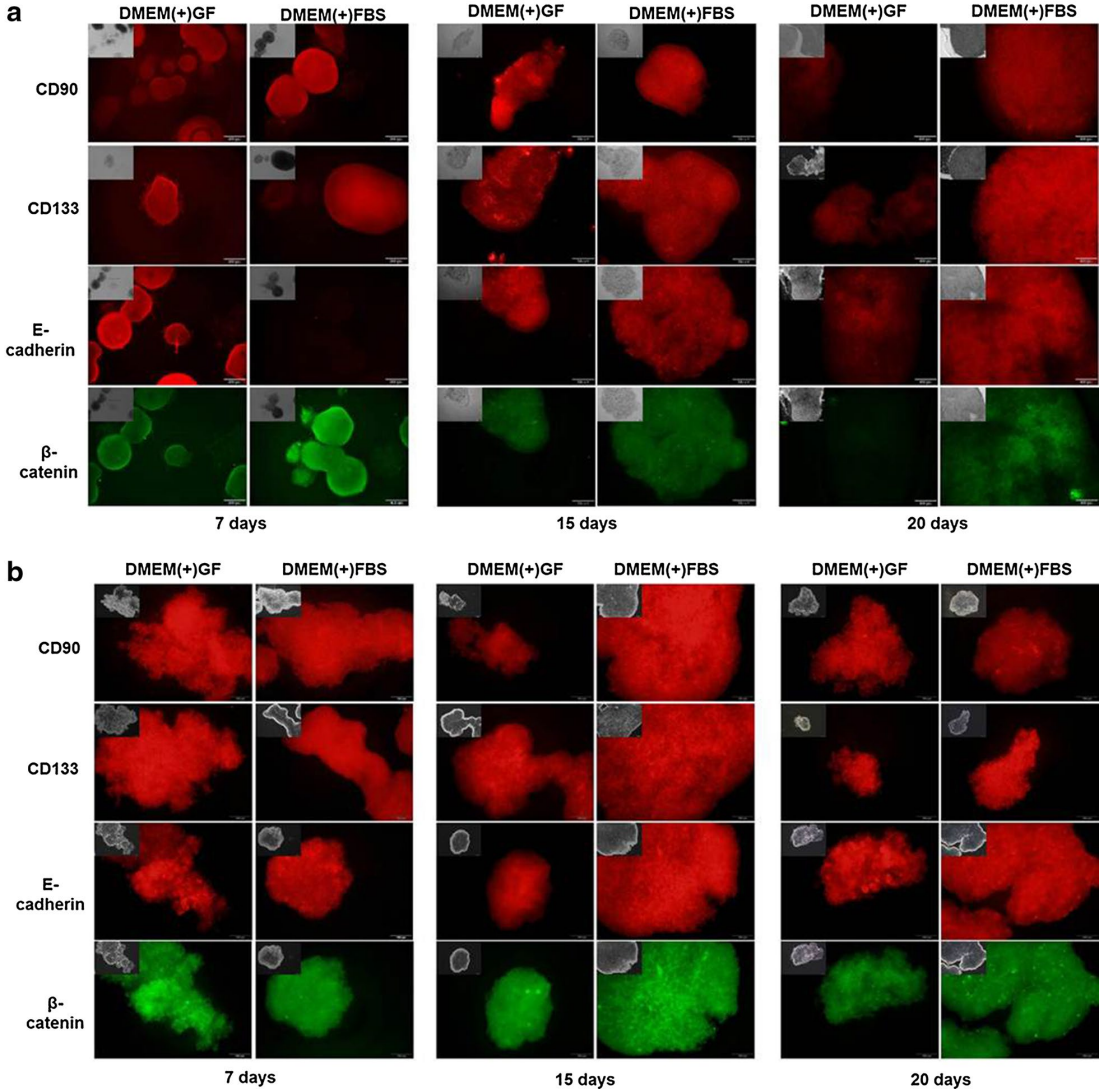
Immunofluorescence staining showed that cancer stem cells markers were highly expressed in Huh7 (**a**) and HepG2 (**b**) sphere-forming cells. Sphere-forming cells showed they were positive for cancer stem cells markers of CD90 (*red*), CD133 (*red*), E-cadherin (*red*), β-catenin (*green*). *Scale bar* 100 µm

Cancer stem cells surface markers CD133 and CD90 were both expressed at two culture media. Beta-catenin, which is involved in the proliferation of CSCs, was expressed on days 7 and 15, but the cells in DMEM(+)FBS expressed relatively more β-catenin on day 20. E-cadherin, which is involved in the physical association of cells, was hardly expressed in DMEM(+)FBS cells on day 7, but was expressed at two culture media on days 15 and 20. In HepG2 sphere-forming cells, CSC surface markers were expressed in both media. Because there was almost no difference between the expression of stem cell markers of cells grown in the DMEM(+)GF and DMEM(+)FBS, it can be inferred that growth factors did not influence these characteristics of the cells (Fig. 3).

### Pluripotency markers expressed in Huh7 and HepG2 sphere-forming cells

Oct4, Nanog, and Sox2 are pluripotency markers. The number of cells expressing each marker was small on day 7, but gradually increased after day 15. On day 20, cells in both the DMEM(+)GF and DMEM(+)FBS expressed these markers similarly.

**Fig. 4:**
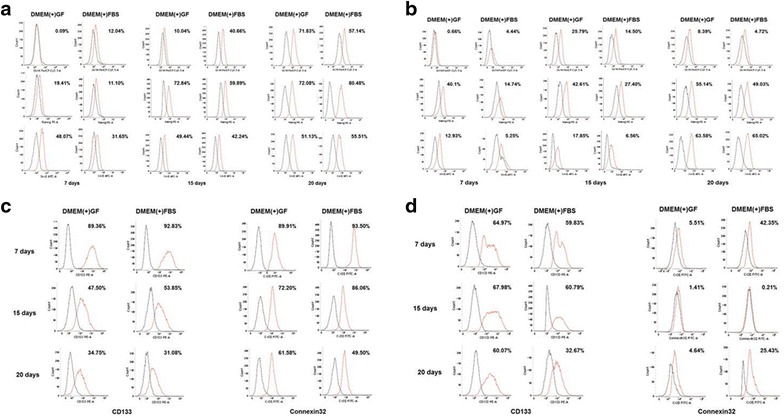
Expression of pluripotency and cancer stem cells markers in sphere-forming cells. Oct4, Nanog, Sox2 were increased similar to the number of Huh7 (**a**) and HepG2 (**b**) sphere-forming cells in both media. **c** Huh7 sphere-forming cells were positive for CD133, Connexin 32. **d** HepG2 sphere-forming cells were positive for CD133 and Connexin 32

Stem cell markers were not expressed early in sphere formation; however, as the culture time progressed, proliferation and the number of cells expressing Oct4, Nanog, and Sox2 were increased. In addition, because stem cell marker expression was similar between the DMEM(+)GF and DMEM(+)FBS-cultured cells, it can be concluded that growth factors do not affect the expression of these genes under these conditions (Fig. 4a, b).

### Cancer stem cell markers expressed in Huh7 and HepG2 sphere-forming cells

The expression levels of Connexin 32 and CD133 were measured by flow cytometry. In Huh7 sphere-forming cells, DMEM(+)GF and DMEM(+)FBS cultured cells exhibited similar levels of Connexin 32 expression, which decreased over time. The ratio of Connexin 32 presenting cells in HepG2 sphere-forming cells was small compared to the ratio of Connexin 32 cells in the Huh7 sphere-forming cells. However, the number of total presenting cells decreased as the days of culture increased.

The expression of CD133, a marker of CSCs, was measured on days 7 and 15 of sphere-forming culture, and the results were similar under the two culture media. On day 20, cells under both media conditions showed a decreased amount of cells expressing CD133 (Fig. 4c, d). Therefore, these data suggest that sphere-forming culture without growth factors is possible without significantly affecting cell pluripotency and proliferation.

## Discussion

Sphere-forming culture methods, in which cells are cultured in a suspended state, have been used to proliferate adult stem cells such as prostate and mammary cells [18, 19]. In the current study, CSC sphere formation was compared between culture conditions including medium with growth factors and with FBS. Spheres formed well under both media conditions, and the characteristics of CSCs were not significantly different between the two media conditions. The proliferation rate of the cells in DMEM(+)GF was higher than that of cells in DMEM(+)FBS after 3 days in culture. It is possible that cells proliferated more rapidly in DMEM(+)GF due to the influence of the added growth factors. However, because the difference in cell proliferation between the DMEM(+)GF and DMEM(+)FBS was minimal after 15 days, we hypothesized that growth factors may affect proliferation initially but less so subsequently. Additionally, both groups had low proliferation rates as the culture period was extended, indicating that apoptosis occurred. Cells in DMEM(−)FBS did not form spheres or proliferate, and most of the cells died in culture. Therefore, DMEM(−)FBS medium was excluded from further experiments.

The expression levels of CSC and stem cell markers were not significantly different between the DMEM(+)GF and DMEM(+)FBS conditions. Pluripotent stem cell markers were minimally expressed at the beginning of culture but increased after days 15 and 20 in both experimental groups. Oct4, Nanog, and Sox2 expression was evaluated, and the results showed no significant difference between DMEM(+)GF and DMEM(+)FBS cultured cells. Oct4 and Nanog were highly expressed in DMEM(+)GF cells at the beginning of culture. Cells cultured in DMEM(+)FBS did not express Oct4 and Nanog early in culture; however, over time, this expression increased to be similar to that in DMEM(+)GF-cultured cells. From these results, we concluded that addition of growth factors does not affect the pluripotency characteristics of cells over time. EpCAM is a prognostic factor used as a biomarker for early HCC that is also expressed in stem cells [20]. EpCAM was expressed strongly in both experimental groups, indicating further that the cells maintained characteristics of CSCs in culture.

β-catenin is a signaling molecule involved in the proliferation and differentiation of hepatoblasts. Increased expression of β-catenin results in increased proliferation and growth of hepatoblastoma and HCC cells [21]. In the current experiment, cells grown under both media conditions expressed β-catenin, indicating cellular proliferation.

Connexin 32 and CD133 are widely used stem cell markers that are identified in cancer tissues such as those of the liver, brain and pancreas to promote tumor metastasis and CSC expansion and self-renewal [11, 22].

DMEM(+)GF and DMEM(+)FBS cultured cells showed expression of Connexin 32 at similar levels. In addition, flow cytometry revealed that the number of sphere-forming cells expressing CD133 was not different between the two media conditions. The HCC marker CD90 was also expressed in both groups, suggesting that the cells may have properties similar to CSCs causing carcinogenesis.

In the both cell lines, the ratio of cells expressing CD133 greatly decreased on day 20. This result suggests that CD133 expression cells can be differentiated, which is a typical characteristic of stem cell line. The continuous addition of growth factor and serum have turned CD133 expressing cells into non-CD133 expressing cells. Previous studies have reported that in the hepatoma cell k\line CD133 and EpCAM expression cells differentiated into non-CD133 and non-EpCAM expressing cells as the days of culture increased [23]. We have shown that sphere-forming culture of CSCs can be performed in growth factor-free medium supplemented with serum with the cells maintaining characteristics of CSCs. Our results suggest that the addition of growth factors does not affect the characteristics or proliferation of CSCs in long-term culture. Sphere-forming CSCs in solid cancer tissues are known to proliferate in serum-free medium supplemented with growth factors such as EGF and bFGF [12, 13]. However, in MCF7, a breast cancer cell line, and A549, a lung cancer cell line, autocrine signaling in response to EGF increased in medium without these growth factors, and EGF receptors were activated to promote and increase CSC sphere formation. Conversely, it was found that suppression of bFGF increased CSC sphere formation [12].

Therefore, analysis of autocrine factors that may affect cell growth and proliferation is needed. Lastly, to identify the differentiation of CSCs into cancer cells, comparison of tumorigenesis by transplanting sphere-forming cells into animal models may be needed.

In conclusion, this study provides evidence that sphere-forming CSCs can grow in culture without growth factors and maintain pluripotency. This finding may help reduce patients’ financial burden given that sphere-forming culture of CSCs may be conducted at a lower cost.

## Methods

### Cell culture

Human hepatocellular carcinoma cells (Huh7, HepG2) were obtained from Korean Cell Line Back (Seoul, Korea). Cell lines (Huh7, HepG2) were cultured at a density of 10,000 cells/cm^2^ in Dulbecco’s Modified Eagle Medium (DMEM) high-glucose medium supplemented with 10 % inactivated fetal bovine serum (FBS), 0.01 % antibiotic–antimycotic (Invitrogen, Grand Island, NY, USA), and incubated at 37 °C with 5 % CO_2_.

### Sphere-forming culture

When cell confluence reached 80 %, the cells were detached using 1× trypsin–EDTA. Cells free from serum were suspended in DMEM/F12 medium supplemented with 1 % b27 supplement, 0.01 % antibiotic–antimycotic (Invitrogen, Grand Island, NY, USA), 20 ng/mL epidermal growth factor [EGF (Invitrogen, Grand Island, NY, USA)], and 20 ng/mL basic fibroblast growth factor [bFGF (Invitrogen, Seoul, Korea)] (DMEG(+)GF), DMEM-high glucose with FBS(DMEM(+)FBS and DMEM-high glucose without FBS(DMEM(−)FBS). Cells were subsequently cultured in ultra-low attachment 24-well plates (24 well plate coated with Ultra-Low Attachment Surface, Corning, NY, USA) at a density of 10,000 cells per well, and were incubated at 37 °C with 5 % CO_2_.

### Cell morphology

After sphere-forming culture, a photo of each media condition was observed with a microscope on days 3, 7, 15, and 20 to observe cell morphology.

### Cell proliferation assay

Cell proliferation rates were measured for cells in each medium type with an enzyme-linked immunosorbent assay (ELISA) reader using Cell Counting Kit-8 from Dojindo Laboratories (Kumamoto, Japan). Cell density was measured at an absorbance of 450 nm on days 3, 7, 15, and 20.

### Reverse transcription-polymerase chain reaction (RT-PCR)

Cells were collected from each culture medium type at days 3, 7, 15, and 20. Total mRNA was extracted from the cells using TRIzol reagent (Invitrogen, CA, USA). cDNA was produced from 2 μg of RNA using RT-PCR, and PCR amplification was performed. Gene expression was visualized using 1.6 % agarose gel electrophoresis. RT-PCR primer sequences are described in Table 1. The degree of expression for each marker was compared using Image J (National Institutes of Health, Bethesda, MD, USA).

**Table 1:** Primer used for RT-PCR

Gene name		Primer sequence	Product size (bp)	Accession no.	Annealing temperature	Number of cycles
OCT 4	S	5′-CGT GAA GCT GGA GAA GGA GAA GCT-3′	245	AF268617	60	34
	A	5′-CAA GGG CCG CAG CTC ACA CAT GTT C-3′				
NANOG	S	5′-CAA AGG CAA ACA ACC CAC TT-3′	394	NM_024865	60	34
	A	5’-ATT GTT CCA GGT CTG GTT GC-3′				
SOX 2	S	5′-AAC CCC AAG ATG CAC AAC TC-3′	100	BC13923.2	60	34
	A	5′-CGG GGC CGG TAT TTA TAA TC-3′				
EpCAM	S	5′-CTG GCC GTA AAC TGC TTT AG-3′	182	BC014785.1	60	34
	A	5′-AGC CCA TCA TTG TTC TGG AG-3′				
Connexin 32	S	5′-GTT TGA GGC CGT CTT CAT GT-3′	188	BC039198.1	60	34
	A	5′-CCA CAT TGA GGA TGA TGC AG-3′				
Connexin 43	S	5′-GGA CAT GCA CTT GAA GCA GA-3′	103	BC026329.1	60	34
	A	5′-GAT GAT GTA GGT TCG CAG CA-3′				
GAPDH	S	5′-TCC ATG ACA ACT TTG TGA TC-3′	452	NM_002046	55	34
	A	5′-TGT AGC CAA ATT CGT TGT TA-3′				

### Immunocytochemistry

Cells were collected from each medium type at days 7, 15, and 20. Cells were washed in phosphate-buffered saline (PBS) and immediately fixed in 10 % formaldehyde solution. Each cell sample was exposed to CD133, CD90, E-cadherin, and beta-catenin primary antibodies. After a 24-h incubation, the anti-rabbit IgG HRL-F (ab) and 2-PE anti-goat IgG-FITC (Santa Cruz, TX, USA) secondary antibodies were attached. Antibodies are described in Table 2. Immunostained cells were observed with a fluorescence microscope.

**Table 2:** Antibodies used for immunocytochemistry

First antibody (1:300 dilution)	Secondary antibody (1:300 dilution)
Rabbit anti-human E-cadherin antibody (Santa Cruz)	Anti-rabbit IgG HRL-F(ab)2-PE (Santa Cruz)
Rabbit anti-human CD90 antibody (Santa Cruz)	Anti-rabbit IgG HRL-F(ab)2-PE (Santa Cruz)
Goat anti-human beta-catenin antibody (Santa Cruz)	Anti-goat IgG-FITC (Santa Cruz)
Goat anti-human CD133 antibody (Santa Cruz)	Anti-goat IgG-FITC (Santa Cruz)

For confocal microscopy, cells were fixed in glass chamber slides with 10 % paraformaldehyde in PBS for 30 min at room temperature. After two washes with PBS, cells were permeabilized with 100 % methanol for 5 min at room temperature. Cells were washed twice with PBS and blocked for 45 min in 5 % bovine serum albumin (BSA) in PBS. Confocal microscopy studies were carried out using a laser-scanning microscope from OLYMPUS (Tokyo, Japan).

### Flow cytometry

The sphere-forming cells were separated into single cells using 1× trypsin–EDTA, centrifuged to precipitate, and cleansed with 0.5 % BSA and 2 mM EDTA in PBS. PE-conjugated antihuman CD133/2 monoclonal antibody (mAb) (Miltenyi Biotec, Bergisch Gladbach, Germany) was then added. After acquiring a cell pellet with the method described above, the primary antibody specific to Connexin 32 was incubated with the pellet at 4 °C for 24 h. The secondary antibody was incubated at room temperature for 1 h, and the fluorescence was measured using flow cytometry. BD Human pluripotent stem cell transcription factor analysis kit (BD Biosciences, San Jose, CA, USA) was used to measure the pluripotency markers Oct4, Nanog, and Sox2.

## Abbreviations

Oct4: octamer-binding transcription factor 4
Sox2: SRY (sex determining region Y)-box 2
EpCAM: epithelial cellular adhesion molecule
GAPDH: glyceraldehyde 3-phosphate dehydrogenase
